# Evaluation of Brain Nuclear Medicine Imaging Tracers in a Murine Model of Sepsis-Associated Encephalopathy

**DOI:** 10.1007/s11307-018-1201-3

**Published:** 2018-05-07

**Authors:** Dávid Szöllősi, Nikolett Hegedűs, Dániel S. Veres, Ildikó Futó, Ildikó Horváth, Noémi Kovács, Bernadett Martinecz, Ádám Dénes, Daniel Seifert, Ralf Bergmann, Ondřej Lebeda, Zoltán Varga, Zoltán Kaleta, Krisztián Szigeti, Domokos Máthé

**Affiliations:** 1 CROmed Translational Research Centers, Budapest, H-1047 Hungary; 2 Department of Biophysics and Radiation Biology, Semmelweis Univ, Budapest, H-1094 Hungary; 3 Laboratory of Neuroimmunology, Institute of Experimental Medicine, Budapest, Hungary; 4 Helmholz-Zentrum Dresden-Rossendorf, Radiopharmazie Radiopharmaceutische Biologie, Dresden, Germany; 5 Nuclear Physics Institute of the CAS, CZ 250 68 Rez, Czech Republic; 6 Biological Nanochemistry Research Group, Institute of Materials and Environmental Chemistry, Research Centre for Natural Sciences, Hungarian Academy of Sciences, Budapest, Hungary; 7 Progressio Fine Chemical Engineering Ltd, Székesfehérvár, Hungary

**Keywords:** Systemic infection, Neuroinflammation, Microglia activation, LPS, [^99m^Tc]HMPAO, [^18^F]FDG, [^125^I]iomazenil, [^125^I]CLINME, SPECT/CT, PET/MRI

## Abstract

**Purpose:**

The purpose of this study was to evaluate a set of widely used nuclear medicine imaging agents as possible methods to study the early effects of systemic inflammation on the living brain in a mouse model of sepsis-associated encephalopathy (SAE). The lipopolysaccharide (LPS)-induced murine systemic inflammation model was selected as a model of SAE.

**Procedures:**

C57BL/6 mice were used. A multimodal imaging protocol was carried out on each animal 4 h following the intravenous administration of LPS using the following tracers: [^99m^Tc][2,2-dimethyl-3-[(3E)-3-oxidoiminobutan-2-yl]azanidylpropyl]-[(3E)-3-hydroxyiminobutan-2-yl]azanide ([^99m^Tc]HMPAO) and ethyl-7-[^125^I]iodo-5-methyl-6-oxo-4H-imidazo[1,5-a][1,4]benzodiazepine-3-carboxylate ([^125^I]iomazenil) to measure brain perfusion and neuronal damage, respectively; 2-deoxy-2-[^18^F]fluoro-d-glucose ([^18^F]FDG) to measure cerebral glucose uptake. We assessed microglia activity on another group of mice using 2-[6-chloro-2-(4-[^125^I]iodophenyl)-imidazo[1,2-a]pyridin-3-yl]-*N*-ethyl-*N*-methyl-acetamide ([^125^I]CLINME). Radiotracer uptakes were measured in different brain regions and correlated. Microglia activity was also assessed using immunohistochemistry. Brain glutathione levels were measured to investigate oxidative stress.

**Results:**

Significantly reduced perfusion values and significantly enhanced [^18^F]FDG and [^125^I]CLINME uptake was measured in the LPS-treated group. Following perfusion compensation, enhanced [^125^I]iomazenil uptake was measured in the LPS-treated group’s hippocampus and cerebellum. In this group, both [^18^F]FDG and [^125^I]iomazenil uptake showed highly negative correlation to perfusion measured with ([^99m^Tc]HMPAO uptake in all brain regions. No significant differences were detected in brain glutathione levels between the groups. The CD45 and P2Y12 double-labeling immunohistochemistry showed widespread microglia activation in the LPS-treated group.

**Conclusions:**

Our results suggest that [^125^I]CLINME and [^99m^Tc]HMPAO SPECT can be used to detect microglia activation and brain hypoperfusion, respectively, in the early phase (4 h post injection) of systemic inflammation. We suspect that the enhancement of [^18^F]FDG and [^125^I]iomazenil uptake in the LPS-treated group does not necessarily reflect neural hypermetabolism and the lack of neuronal damage. They are most likely caused by processes emerging during neuroinflammation, *e.g.*, microglia activation and/or immune cell infiltration.

**Electronic supplementary material:**

The online version of this article (10.1007/s11307-018-1201-3) contains supplementary material, which is available to authorized users.

## Introduction

Sepsis-associated encephalopathy (SAE) is a devastating complication of severe acute systemic inflammation. It causes both acute and long-lasting neurological dysfunction and contributes to the mortality of patients with sepsis [1]. Current clinical approaches are mainly based on the earliest possible diagnosis and treatment of the systemic inflammation, but our knowledge of the pathophysiological processes overwhelming the brain at this early stage of sepsis is far from complete. Understanding these processes could lead to the development of disease-specific diagnostic and therapeutic approaches that could potentially protect the brain from systemic inflammation and improve mortality.

Much of our current knowledge of SAE has been gathered from animal studies [2]. One of the most important animal models is the lipopolysaccharide (LPS)-induced murine systemic inflammation model. Following the systemic administration of LPS, the mouse brain exhibits a variety of acute and long-lasting alterations including the elevation of inflammatory cytokines [3–7], microglia activation [8, 9], neuron damage [3], altered neurotransmission [10], oxidative stress [3, 11], blood-brain barrier changes [3, 12] vascular adhesion [13], or invasion of immune cells [14]. Similarities have been found between this mouse model and human SAE [12, 15–18], making it also a model of murine SAE. A favorable approach to investigating the brain during systemic inflammation is multimodal nuclear medicine imaging [19, 20]. This approach could provide a means to investigate the little-known spatiotemporal distribution and correlations of multiple parameters related to pathophysiology. Brain region-specific connections between the pathophysiologic processes also provide important implications for neuroinflammation in general.

Even if a radiopharmaceutical is highly specific to a certain target, its biodistribution may not be dependent on a single biological process. In turn, many different pathophysiological factors can influence uptake by the specified target (*e.g.*, an increase in 2-deoxy-2-[^18^F]fluoro-d-glucose ([^18^F]FDG) uptake could be caused by a wide variety of processes) [21]. Parameters measured in healthy brain or during neuroinflammation could be determined by quite different disease-specific processes.

The aim of this study was to assess whether quantitative multimodal *in vivo* imaging with a set of widely used radiotracers (Table 1) could be used to investigate a set of brain alterations and their region-specific connections associated to the early phase of neuroinflammation induced by systemic LPS injection in mice.

**Table 1:** A summary of the radiotracers and modalities used in this study

Radiotracer	Abbreviation	Modality	Putative alteration/process
[^99m^ Tc][2,2-dimethyl-3-[(3E)-3-oxidoiminobutan-2-yl]azanidylpropyl]-[(3E)-3-hydroxyiminobutan-2-yl]azanide	[^99m^Tc]HMPAO	SPECT	Brain perfusion [22]
ethyl 7-[^125^I]iodo-5-methyl-6-oxo-4H-imidazol[1,5-a][1,4]benzodiazepine-3-carboxylate	[^125^I]iomazenil	SPECT	Neuronal damage/apoptosis [23–26]
2-[6-chloro-2-(4-[^125^I]iodophenyl)-imidazo[1,2-a]pyridin-3-yl]-N-ethyl-N-methyl-acetamide	[^125^I]CLINME	SPECT	Microglia activation [27]
2-deoxy-2-[^18^F]fluoro-D-glucose	[^18^F]FDG	PET	Cerebral glucose uptake [28]

SPECT: single photon emission computed tomography, PET: positron emission tomography.

We investigated the following: brain perfusion with [^99m^Tc]HMPAO single photon emission computed tomography (SPECT), brain glucose metabolism with [^18^F]FDG positron emission tomography (PET), neuron damage with the central benzodiazepine receptor ligand [^125^I]iomazenil SPECT, and microglia activation with the 18 kDa translocator protein (TSPO, or, peripheral benzodiazepine receptor, PBR) ligand [^125^I]CLINME SPECT. We described microglia activation with immunohistochemistry (IHC) and oxidation state by a fluorometric *ex vivo* glutathione assay. These methods have been validated for the respective alterations in multiple models (see references in Table 1).

## Materials and Methods

### Summary of the Experiments

**Fig. 1:**
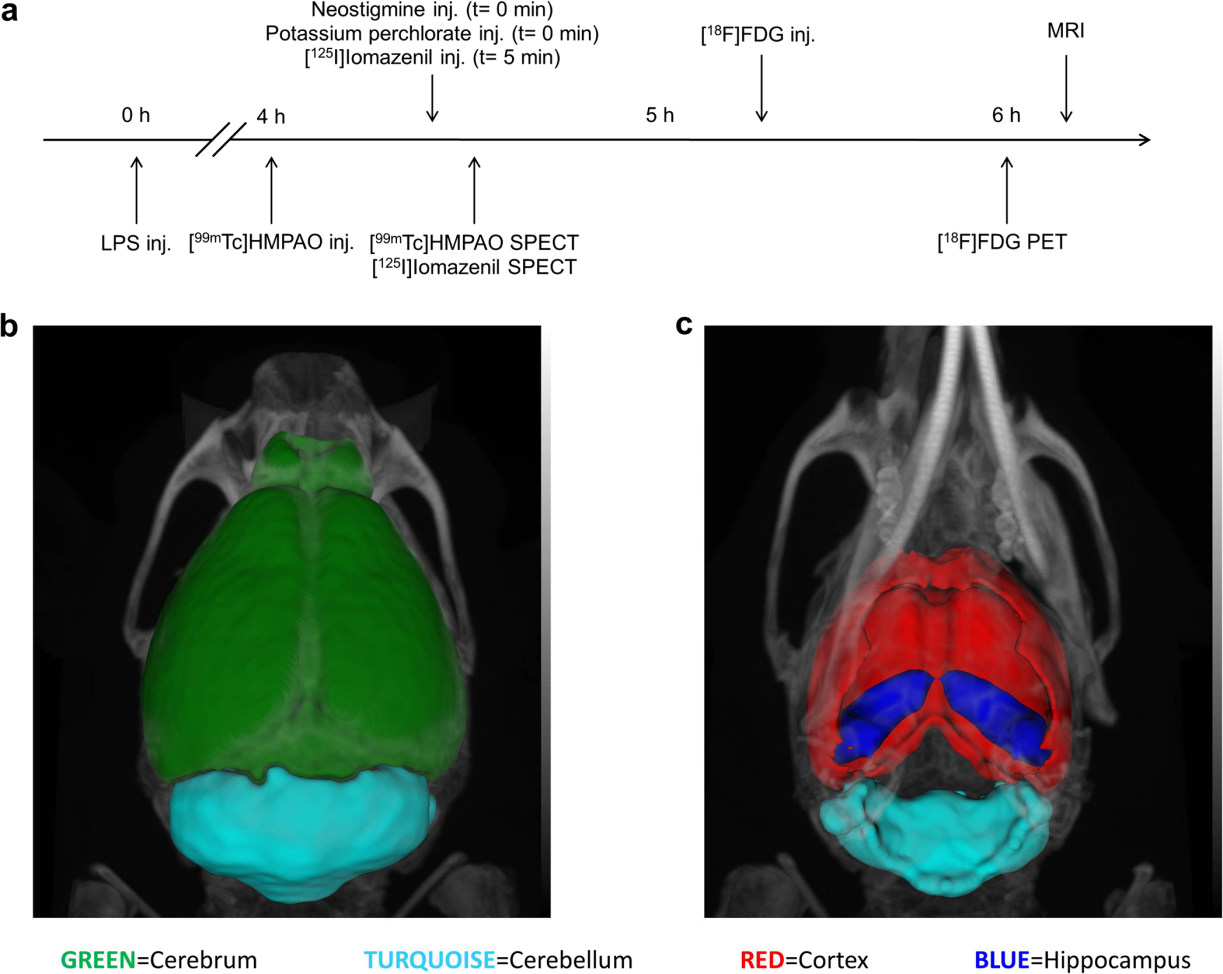
Illustration of the methods. **a** Experimental protocol for *in vivo* measurements. **b** Dorsal view of MRI coregistration with CT showing the segmented 3D brain regions. **c** Ventral view of the same VOIs (volumes of interest). Representing the cerebrum (green: this entity includes the whole brain without cerebellum), cerebellum (turquoise), cortex (red), and hippocampus (blue).

The experiments are summarized in Fig. 1a. [^99m^Tc]HMPAO and [^125^I]iomazenil dual SPECT, and [^18^F]FDG PET were carried out on LPS-treated and control animals and the correlations of the results were computed. These animals were later used for the *ex vivo* glutathione assay. [^125^I]CLINME SPECT and IHC measurements were completed on different animals due to the methodical incompatibility of these assays with previous ones. These two measurements were used to study the variability of brain region-specific microglial response. MR images were used to segment the brain into 3D volumes of interest (cerebrum—indicating the whole brain without cerebellum, cerebellum, cerebral cortex, and hippocampus) using a connected threshold algorithm (Fig. 1b, c).

Glutathione levels were determined *ex vivo* using a colorimetric assay. Microscopically, resting (highly ramified, P2Y12+ cells with low CD45 signal [29–32]) and activated (P2Y12+, CD45_low_ ramified cells with thickened processes and enlarged body) microglia cells were counted. Blood-derived leukocytes (CD45-positive, round shape cells with predominantly perivascular location) [32, 33] were excluded from analysis. The correlation coefficients of measured nuclear medicine parameters per brain regions *in vivo* were calculated with correlation analysis (GraphPadPrism6.0, GraphPad Software Inc., La Jolla, CA, USA).

Experimental details are further described in the Electronic Supplementary Material (ESM) under the “Materials and Methods” section.

### Perfusion Compensation and Data Analysis

For perfusion compensation [^125^I]iomazenil uptake was divided by the same animals’ simultaneously measured [^99m^Tc]HMPAO uptake in each region to eliminate the inflammation-related relative blood flow changes. Normality of data sets was assessed with the Kolmogorov-Smirnov test. Data from *in vivo* measurements (PET and SPECT scans) were analyzed with the one-sided permutation test. This test is a conditional statistical procedure where the conditioning is with respect to the observed data set [34]. The correlation coefficients per brain regions were calculated with correlation analysis. Data from immunohistochemical studies were analyzed with unpaired *t* tests (GraphPadPrism6.0, GraphPad Software Inc., La Jolla, CA, USA). In all cases, *p* value ≤ 0.05 was considered as statistically significant.

## Results

### [^99m^Tc]HMPAO SPECT Imaging

**Fig. 2:**
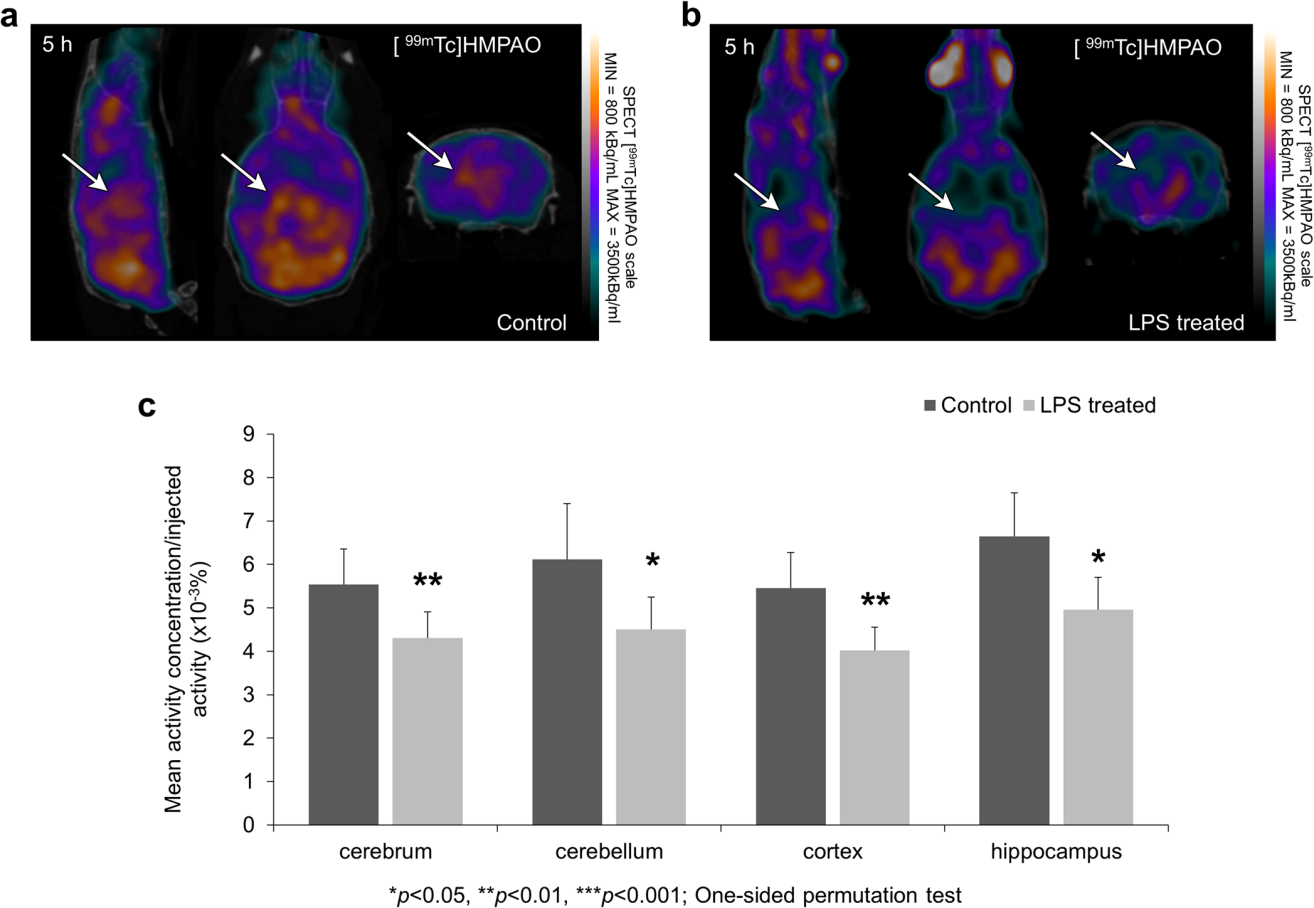
SPECT imaging reveal decreased perfusion after LPS injection. Cerebral blood perfusion was measured by [^99m^Tc]HMPAO. SPECT coregistration with computed tomography (CT) showing [^99m^Tc]HMPAO uptake in **a** control and **b** LPS-treated animals. Arrows indicate areas where the difference in radiotracer uptakes between the two groups is observable. **c** [^99m^Tc]HMPAO uptake is significantly reduced 5 h after the LPS injection in all examined brain regions (cerebrum: indicates the whole brain without cerebellum, cerebellum, cortex, and hippocampus; *p ≤ 0.05; **p < 0.01; ***p < 0.001—one-sided permutation test).

The results of [^99m^Tc]HMPAO SPECT measurements are illustrated in Fig. 2a, b. In every segmented brain region (cerebrum, cerebellum, cerebral cortex, and hippocampus), significantly reduced (*p* < 0.05) [^99m^Tc]HMPAO uptake was observed in the LPS-treated group compared to the control (Fig. 2c).

### [^125^I]iomazenil-SPECT Imaging

**Fig. 3:**
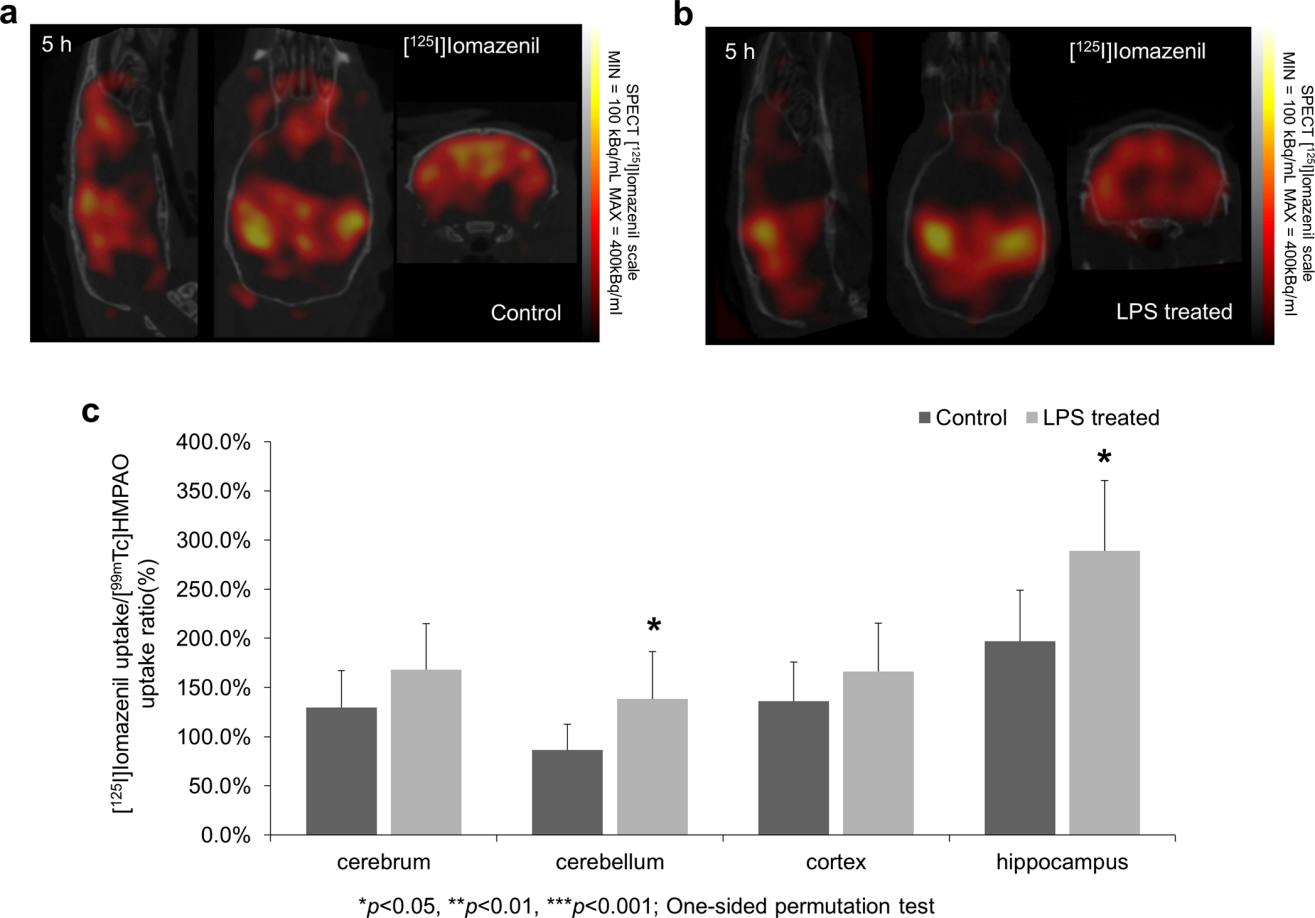
SPECT imaging of [^125^I]iomazenil following LPS injection. SPECT coregistration with CT showing iomazenil uptake in **a** control and **b** LPS-treated animals. **c** [^125^I]iomazenil uptake is significantly increased 5 h after the LPS injection in cerebellum and hippocampus (*p ≤ 0.05—one-sided permutation test). Relevant changes were also observed and measured in the area of cerebrum and cortex but these differences were not significant (*p* = 0.095, *p* = 0.138, respectively).

The results of [^125^I]iomazenil SPECT measurements are illustrated in Fig. 3a, b. Perfusion compensation resulted in significantly enhanced [^125^I]iomazenil uptake values in the LPS-treated group’s cerebellum and hippocampus compared to the control. Relevant changes were seen in the cortex and the whole cerebrum but these differences were not significant (Fig. 3c).

### [^18^F]FDG PET Imaging

**Fig. 4:**
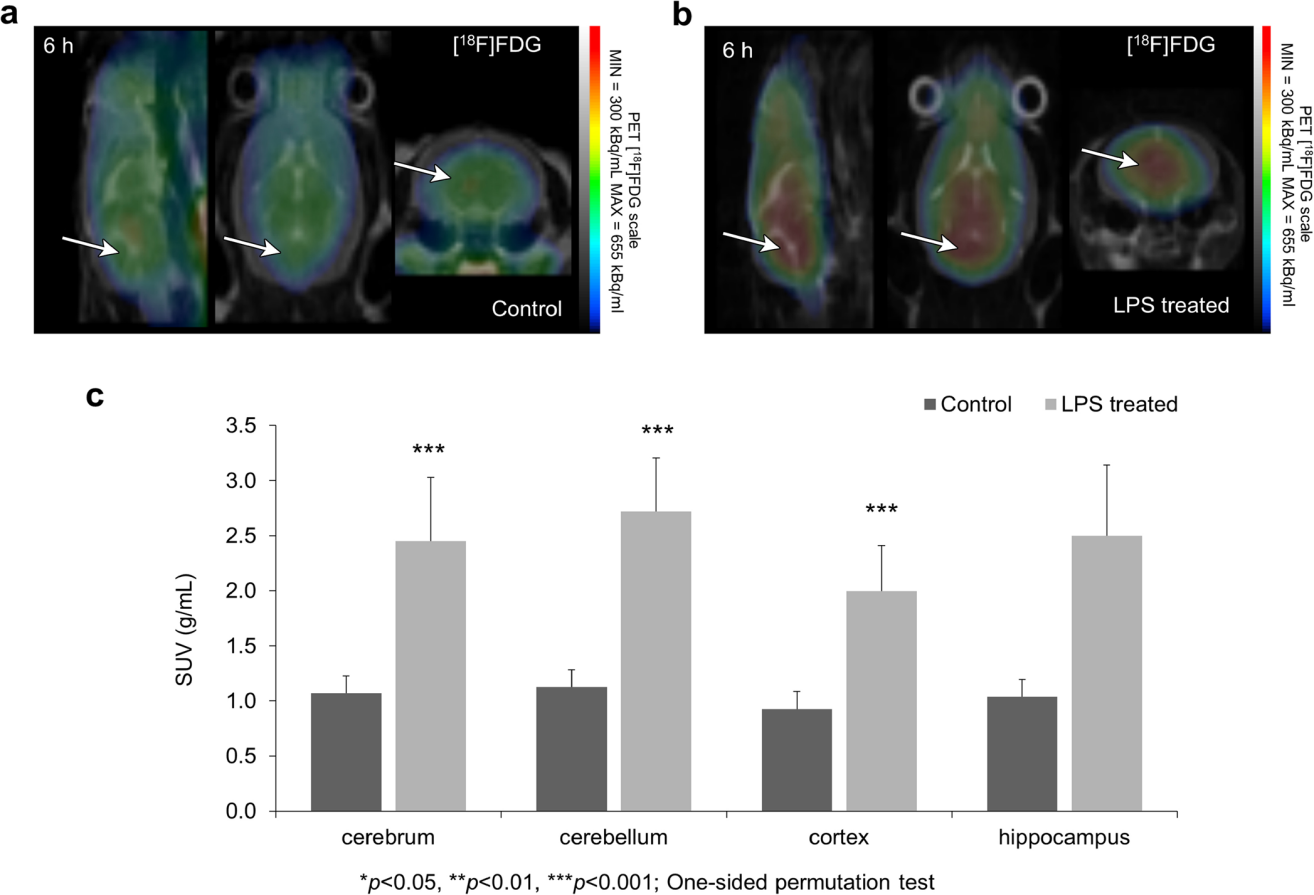
PET imaging after LPS injection. Cerebral glucose transport and metabolism was measured by [^18^F]FDG. Summarized PET signal during a 3 min time frame starting at 7 min post injection and ending at 10 min post injection of [^18^F]FDG is co-registered with CT showing [^18^F]FDG uptake in **a** control and **b** LPS-treated animals. Arrows indicate example areas where the difference in radiotracer uptakes between the two groups is visually discernable. **c** [^18^F]FDG uptake is significantly increased 6 h after the LPS injection in cerebrum—defined as the whole brain without the cerebellum, cerebellum, and cortex. Relevant but not significant changes were registered in hippocampus (*p* = 0.057) (***p < 0.001—one-sided permutation test).

[^18^F]FDG measurements were able to visualize early changes of metabolic activity following LPS injection (Fig. 4a, b, Supplementary Fig. 1). In almost all segmented brain regions (cerebrum, cerebellum, and cerebral cortex), significantly enhanced (*p* < 0.05) [^18^F]FDG uptake was measured in the treated group compared to the control (Fig. 4c).

### [^125^I]CLINME-SPECT Imaging

**Fig. 5:**
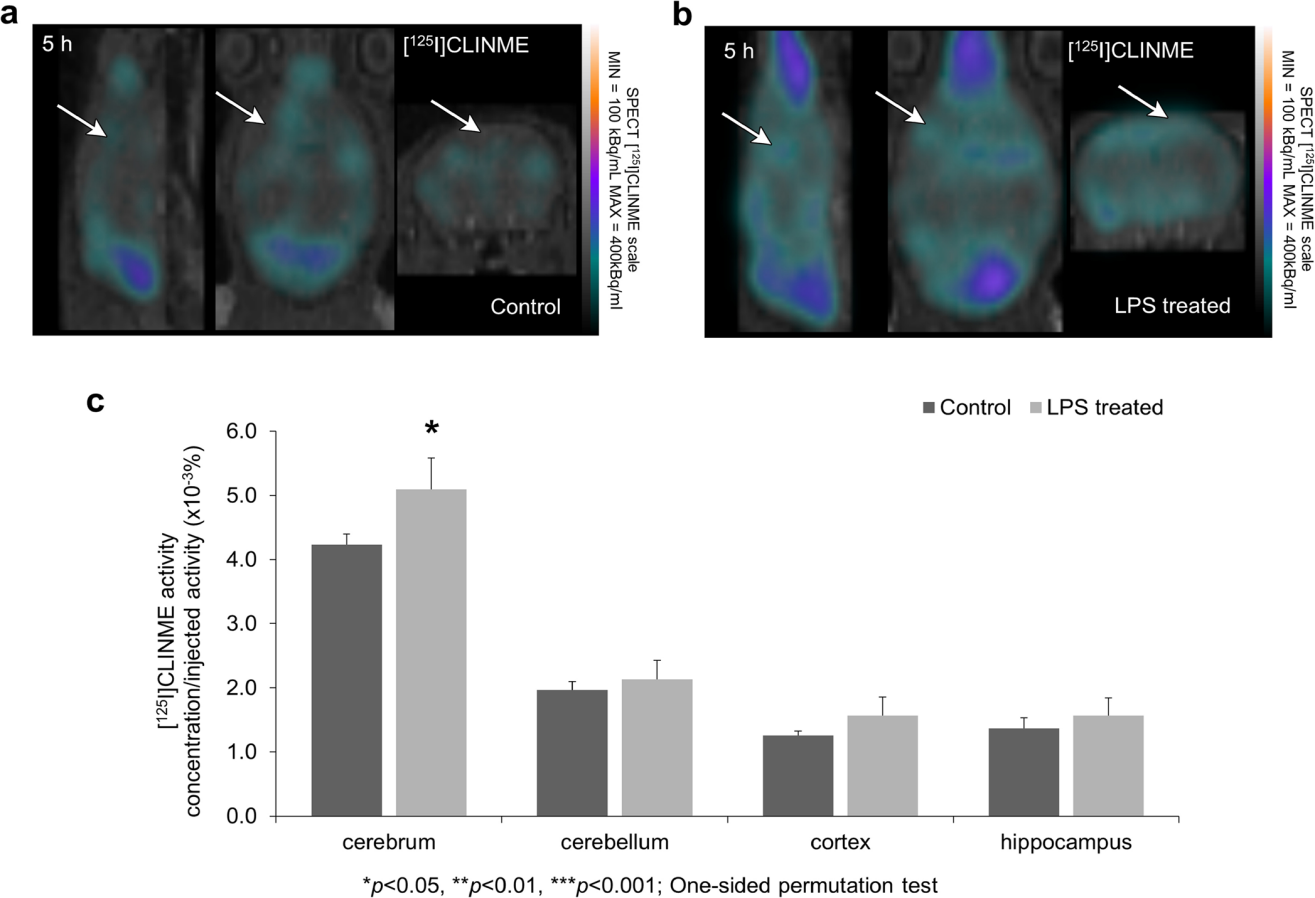
Microglia activation was indirectly measured by [^125^I]CLINME uptake. SPECT coregistration with CT showing [^125^I]CLINME uptake changes after **a** LPS-induced neuroinflammation compared to **b** the control group. Arrows indicate example areas where the difference in radiotracer uptakes between the two groups is visually discernable. **c** [^125^I]CLINME uptake is significantly increased 5 h after the LPS injection in the cerebrum (*p ≤ 0.05—one-sided permutation test).

[^125^I]CLINME SPECT results are shown in Fig. 5a, b. Significantly enhanced (*p* = 0.05) uptake was observed in the cerebrum and marked, but not significant elevation in all other investigated brain areas (Fig. 5c).

### Correlation Studies

The results of the correlation studies are listed in Table 2 and illustrated in Supplementary Fig. 2. In the LPS-treated group, highly positive correlation was found between the uptake values of [^18^F]FDG and [^125^I]iomazenil while these values had a strong negative correlation with [^99m^Tc]HMPAO uptake in all investigated regions. In the control group, strong negative correlation coefficients were found between the uptake of [^18^F]FDG and [^125^I]iomazenil in the cerebrum, cortex, and hippocampus, while small positive correlation coefficients were detected in the cerebellum. This brain region showed highly negative correlation between the uptake values of [^125^I]iomazenil and [^99m^Tc]HMPAO. Moderate negative correlations were found between [^18^F]FDG and [^99m^Tc]HMPAO uptake values in the cortex and cerebellum.

**Table 2:** The average correlation coefficients in LPS treated and control groups.

Brain region	Correlated tracer uptake values	Control	LPS treated
Cerebrum	[^18^F]FDG/[^125^I]iomazenil	− 0.7023	0.9419
	[^18^F]FDG /[^99m^Tc]HMPAO	− 0.2578	− 0.9859
	[^125^I]iomazenil/[^99m^Tc]HMPAO	− 0.1907	− 0.9847
Cortex	[^18^F]FDG/[^125^I]iomazenil	− 0.9341	0.9985
	[^18^F]FDG /[^99m^Tc]HMPAO	− 0.5212	− 0.9976
	[^125^I]iomazenil/[^99m^Tc]HMPAO	0.2411	− 0.9925
Hippocampus	[^18^F]FDG/[^125^I]iomazenil	− 0.8004	0.8544
	[^18^F]FDG /[^99m^Tc]HMPAO	− 0.3207	− 0.9621
	[^125^I]iomazenil/[^99m^Tc]HMPAO	− 0.2260	− 0.9636
Cerebellum	[^18^F]FDG/[^125^I]iomazenil	0.2849	0.9775
	[^18^F]FDG /[^99m^Tc]HMPAO	− 0.8212	− 0.8723
	[^125^I]iomazenil/[^99m^Tc]HMPAO	− 0.8212	− 0.7495

*Ex vivo glutathione level measurements showed no significant changes* (*for details see Supplementary Results*)*.*

### Immunohistochemistry

**Fig. 6:**
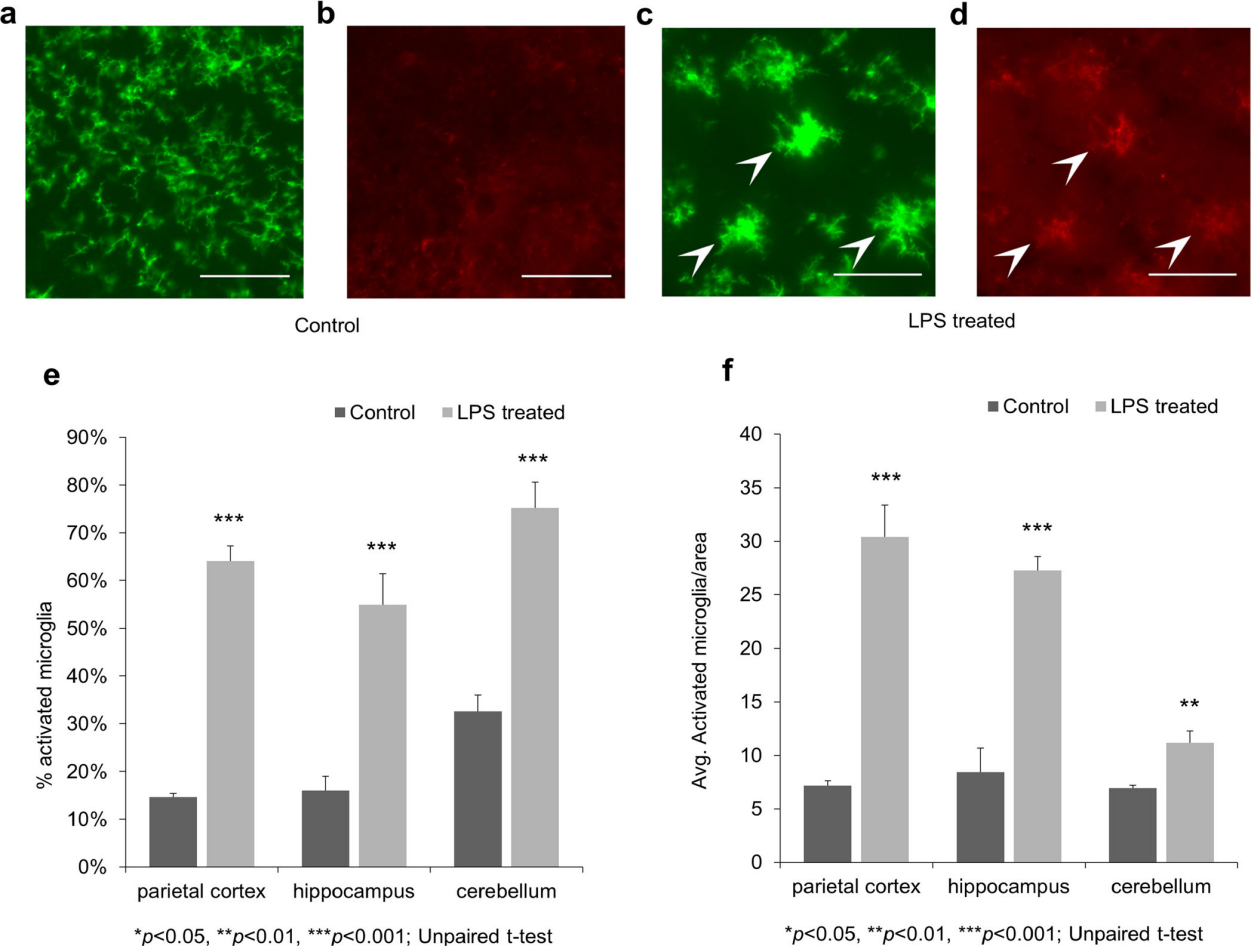
P2Y12 and CD45 double-labeling immunohistochemistry before and after LPS treatment. Representative photomicrographs from the hippocampus. All scale bars correspond to 50 μm. **a** P2Y12 brain immunostaining of control animals reveals ramified, P2Y12+ microglia in all brain regions (parietal cortex, hippocampus, and cerebellum). **b** The CD45 immunostaining of the same area reveals very low CD45 immunoreactivity. **c** P2Y12 staining reveals activated microglia cells with enlarged cell bodies and thickened processes in LPS-treated animals (arrowheads). **d** Double labeling with CD45 shows the CD45^low^ expression of the corresponding cells (arrowheads). **e** The percentage of activated/all microglia based on morphology and CD45 expression. **f** The number of activated microglia per brain area.

The CD45 and P2Y12 double-labeling immunohistochemistry revealed microglial activation in response to systemic inflammation within 4 h after LPS administration (Fig. 6 a–d). Both the percentage of activated/all microglia (Fig. 6e) and the number of activated microglia/area (Fig. 6f) were significantly (*p* < 0.01) higher in the LPS-treated group compared to the control group in all investigated regions.

## Discussion

Tissue hypoperfusion is one of the hallmarks of sepsis syndrome and the brain is not an exception. In humans, decreased perfusion and impaired vascular autoregulation have been reported by multiple authors [17, 35–37]; however, this mechanism seems to be controversial [1]. Our dual SPECT measurement showed reduced [^99m^Tc]HMPAO uptake in the brain of LPS-treated animals. Similar distributions were observed both in the control group and the LPS-treated group but the measured uptake quantities were significantly reduced in the latter (Fig. 2a–c). The decreased perfusion might lead to metabolic imbalance and subsequent early and late phase adaptation of glucose transport and utilization by the brain’s most metabolically active cells, astroglia and neurons.

Cerebral metabolic alterations have been previously suggested in SAE [38]. A decrease in cerebral glucose metabolism measured with [^18^F]FDG-PET after 24 h following LPS injection in rats has previously been reported [39]. In contrast, we have observed an early increase in [^18^F]FDG uptake 5 h following the induction of systemic inflammation in mice (Fig. 4a–c). Significantly enhanced [^18^F]FDG uptake values were observed in the cerebrum, cortex, and cerebellum (*p* < 0.05). Our measurements were carried out on anesthetized mice to avoid introducing additional variability resulting from an awake uptake phase [40]. The opposite alterations in perfusion and [^18^F]FDG uptake could be explained by two mechanisms: neurovascular decoupling or the metabolic activity of microglia and infiltrating immune cells. Decoupling during inflammation has been reported in both human [41] and animal studies [42] but it would not fully explain the rise in [^18^F]FDG uptake we measured. Both SAE and the LPS model leads to an increased microglial activity and the infiltration of peripheral immune cells in the brain. These cells also express glucose transporters and can contribute to [^18^F]FDG PET signal during neuroinflammation [43] making them the most likely cause of the increased [^18^F]FDG uptake we observed.

In order to be able to image two isotopes with SPECT in the same animal at the same time, we used [^125^I]iodine. Mouse imaging with [^125^I]iodine is a well-established quantitative possibility even with minuscule injected activities such as 0.2 MBq per animal [44–47]. For [^125^I]iodine containing radiopharmaceuticals, we used potassium perchlorate to competitively inhibit iodine uptake of different peripheral tissues *via* the sodium iodine symporter (NIS) [48, 49].

Neuronal damage and cell death has been previously described both in human SAE and animal models of sepsis [2]. Neuron loss could be the mechanism leading to long-term cognitive impairment observed in critically ill patients [50]. Radiolabeled iomazenil and flumazenil are widely regarded as nuclear medicine tracers indicating neuronal integrity and neuron loss [51–53]. Surprisingly, our measurements showed that [^125^I]iomazenil, a partial inverse agonist of the central benzodiazepine receptor, has an increased uptake in the brains of LPS-treated mice. (Fig. 3a–c). In a previous study, Parente A. et al. investigated the possibility of experimental neuroinflammation influencing the cerebral pharmacokinetics of [^11^C]flumazenil [54]. They observed no significant differences in radiotracer uptake between control and herpes simplex encephalitis rats. Contrarily, our results suggest that brain [^125^I]iomazenil uptake (a SPECT analogue of [^11^C]flumazenil) can be directly influenced by neuroinflammation during the early phase of systemic inflammation. Several putative mechanisms could contribute to the increased uptake. GABA_A_ receptors are present on microglia [55], astrocytes [56–58], and infiltrating immune cells [59, 60]. Furthermore [^125^I]iomazenil can also bind to the peripheral benzodiazepine receptor (TSPO) with micromolar affinity which has an increased glial expression during neuroinflammation [61]. [^125^I]iomazenil as an ester type molecule can be easily degraded by tissue esterase [62]. The additionally injected neostigmine (cholinesterase enzyme blocker in order to enhance plasma stability of [^125^I]iomazenil) could have increased the availability of [^125^I]iomazenil in the brain making low affinity TSPO binding more likely. Since all of these non-neuronal mechanisms that arise during neuroinflammation can play a role in the measured signal, [^125^I]iomazenil is an unreliable marker of neuronal damage in the LPS model and also possibly other models of sepsis. On the other hand, these results raise important questions regarding the GABA_A_ system during neuroinflammation and a potential role for [^125^I]iomazenil as an immune system-related radiotracer of neuroinflammation.

Various studies have confirmed the presumed role of TSPO as a marker of neuroinflammation [63, 64] based on its up-regulated expression on microglial cells, astrocytes, and increased ligand binding after neural damage [65] but its exact functional role is unknown [66]. In our experiments, we applied [^125^I]CLINME for TSPO imaging. In the LPS-treated group, significantly enhanced (*p* = 0.05) [^125^I]CLINME uptake values were measured in the cerebrum, and a marked, but statistically not significant enhancement in the other brain regions of the treated group (Fig. 5a–c). The lack of significant results is most likely due to the low signal-to-noise ratio of our measurements resulting from the combination of low injected activity and small regions of interest. Due to the larger size of the cerebrum VOI, the noise has a lesser impact on the activity measured there. Elevated TSPO expression in LPS-induced systemic inflammation has also been observed in non-human primates [67] and human subjects [68].

The results of the correlation studies (Table 2) outline that the brain region-specific pairwise correlation of [^125^I]iomazenil, [^99m^Tc]HMPAO, and [^18^F]FDG uptake values is different between the control and LPS-treated group. The brain region dependence of correlation coefficients is much lower in the LPS-treated animals than the controls. In healthy animals, [^18^F]FDG, [^125^I]iomazenil, and [^99m^Tc]HMPAO uptake mostly depends on cerebral glucose metabolism, GABA_A_ receptor density, and cerebral perfusion, respectively. In the LPS-treated animals, the highly positive correlation between [^18^F]FDG and [^125^I]iomazenil uptake in all investigated brain regions suggest that inflammatory processes could indeed influence both of these values as discussed earlier. Further supporting this hypothesis, microglia activation was also significantly elevated regardless of brain region (based on IHC and [^125^I]CLINME SPECT results). The highly negative correlations between [^99m^Tc]HMPAO and [^18^F]FDG or [^125^I]iomazenil also fit into this idea if we assume that cerebral hypoperfusion could indicate the severity of inflammation and thus correlate with the metabolic activity and activation state of microglia and infiltrating immune cells that positively contribute to [^18^F]FDG and [^125^I]iomazenil signal.

As there were no differences in *ex vivo* glutathione state, we presume time course of GSH-GSSG transformation seems to be too quick to separately measure GSH and GSSG levels by the applied Glutathione Detection Kit.

P2Y12 and CD45 double-labeling immunohistochemical (IHC) studies proved the activation of microglia in all the examined brain regions of the LPS-treated animals (Fig. 6). The metabotropic purinergic receptor P2Y12 is expressed by resting and activated microglia which can be used to distinguish them from other CNS cells or myeloid lineage cells (*e.g.*, recruited leukocytes) [69, 70]. Although its expression levels were shown to highly depend on the activation and polarization states of microglia [49, 71], here it was used only to identify them and assess their morphology. CD45 is a cell surface glycoprotein expressed in all nucleated hematopoietic cells [72]. It has been shown that CD45 expression is up-regulated in activated microglia in different diseases and models [73–76]. By assessing the morphology and CD45 immunoreactivity of microglia, we were able to distinguish between activated and resting cells with a high degree of certainty.

## Conclusion

In conclusion, we have described the brain region-specific uptake of a set of widely used radiotracers ([^99m^Tc]HMPAO, [^125^I]iomazenil, [^18^F]FDG) during the early phase of LPS-induced murine systemic inflammation. Our results suggest that inflammatory processes can directly contribute to the uptake of [^125^I]iomazenil and [^18^F]FDG masking the neuroinflammation-induced neuron damage and hypometabolism of neural tissue, respectively. Furthermore, we have showed that [^99m^Tc]HMPAO and [^125I^]CLINME can be used to detect cerebral hypoperfusion and microglia activation, respectively, as early as 4 h following the i.v. injection of LPS. Further investigation of the metabolic activity of different brain cells and the status of the GABA receptor system of infiltrating immune cells would be necessary to determine the exact source of the measured signal differences during the early phase of systemic inflammation.

## Electronic supplementary material

ESM 1 (PDF 314 kb)
