# Supplementary material for: Evaluation of Brain Nuclear Medicine Imaging Tracers in a Murine Model of Sepsis-Associated Encephalopathy
Source: Mol Imaging Biol. 2018 May 7;20(6):952–62. doi: 10.1007/s11307-018-1201-3 (PMC6244542; doi:10.1007/s11307-018-1201-3)
Supplement: Supplementary file 1 — (PDF 314 kb) [file 11307_2018_1201_MOESM1_ESM.pdf]

Electronic Supplementary Material

# Evaluation of Brain Nuclear Medicine Imaging Tracers in a Murine Model of Sepsis Associated Encephalopathy

Journal: Molecular Imaging and Biology

Dávid Szöllősi[2], Nikolett Hegedűs[2], Dániel S. Veres[2], Ildikó Futó[2], Ildikó Horváth[2], Noémi Kovács[1], Bernadett Martinecz[3], Ádám Dénes[3], Daniel Seifert[5], Ralf Bergmann[4], Ondřej Lebeda[5], Zoltán Varga[2,6], Zoltán Kaleta[7], Krisztián Szigeti[2*†], Domokos Máthé[1,2]†

[1]CROmed Translational Research Centers, H-1047 Budapest, Hungary

[2]Department of Biophysics and Radiation Biology, Semmelweis Univ, Budapest, Hungary

[3]Laboratory of Neuroimmunology, Institute of Experimental Medicine, Budapest, Hungary

[4]Helmholz-Zentrum Dresden-Rossendorf, Radiopharmazie Radiopharmaceutische Biologie, Dresden, Germany

[5]Nuclear Physics Institute of the CAS, CZ 250 68 Rez, Czech Republic

[6]Biological Nanochemistry Research Group, Institute of Materials and Environmental Chemistry, Research Centre for Natural Sciences, Hungarian Academy of Sciences, Budapest, Hungary

[7]Progressio Fine Chemical Engineering Ltd, Székesfehérvár, Hungary

† Krisztián Szigeti and Domokos Máthé contributed equally to this work.

*Corresponding author: Krisztián Szigeti

Dep. of Biophysics and Radiat. Biol., Semmelweis Univ., H-1094, Budapest, Hungary
Email address: szigeti.krisztian@med.semmelweis-univ.hu
Tel.: +36 1 459 1500/60239
Fax: +36 1 266 6656

Running head: Brain imaging tracers in systemic inflammation

## Materials and Methods

### *Animals*

Experiments were performed in adult male C57BL/6 mice (control n = 12, body weight = 26.00 ± 5.34 g; LPS-treated n = 10 body weight=27.27 ± 5.84), bred in the Animal House of Semmelweis University. Animals were allowed free access to food and water and maintained under temperature, humidity and light-controlled conditions. All procedures were conducted in accordance with the ARRIVE guidelines and the guidelines set by the European Communities Council Directive (86/609 EEC) and approved by the Animal Care and Use Committee of the IEM and the Semmelweis University (XIV-I-001/29-7/2012).

### *Systemic Inflammation*

The systemic inflammation was induced by 3.3 mg/kg body weight of LPS (serotype: 0111:B4, Sigma-Aldrich, St. Louis, MO, USA, L4391). The injection was administered 4 h before the scans via tail vein injection.

### *[$^{125}$I]CLINME preparation*

CLINME molecules (precursor synthesis by Progressio's own process) were radiolabeled using 195.7 MBq [$^{125}$I]NaI by the classic chloramine-T radiolabeling. Freshly prepared 90 µl 0.3 M phosphate buffered saline (PBS), 60 µl 2mg/ml chloramine-T (Sigma-Aldrich, St. Louis, MO, USA), and 30 µl 0.05 M CLINME (Progressio Budapest, Hungary) were mixed and incubated for 10 min at 90°C. The labeling reaction was stopped by 60 µl 0.1 M $Na_2S_2O_3$ (Sigma-Aldrich, St. Louis, MO, USA) and purified via Sep Pak plus C18 (Waters Corporation, Massachusetts, USA). The final product was released after the successful HPLC quality control procedure (radiochemical purity > 95%). The specific activity of the product was 28.00 GBq/mmol, the activity concentration was 50.00 MBq/ml and the radiochemical purity was 95%.

*In vivo imaging protocol*

Mice were anesthetized with isoflurane strictly adhering to our protocol in all cases (3.5-4 % induction and then reduced to 1.52 % for maintenance of anesthesia during scanning). Before imaging 30.63 ± 5.04 MBq (mean±SD) of [$^{99m}$Tc]HMPAO (specific activity: 7.33 GBq/mg according to the manufacturer, Medi-Radiopharma Co Ltd. Érd, Hungary) and 1.92±0.03 MBq (mean±SD) of ultrapure [$^{125}$I]iomazenil (specific activity: 21.00 GBq/µmol, radiochemical purity: 96.50% Nuclear Physics Institute, Rez, Czech Republic) were administered intravenously. Immediately before [$^{125}$I]iomazenil administration 5 mg/kg body weight of neostigmine (Sigma-Aldrich, St. Louis, MO, USA) was administered intravenously and 14 mg/kg body weight of potassium perchlorate (Sigma-Aldrich, St. Louis, MO, USA) was injected subcutaneously. Regional SPECT/computed tomography (CT) was followed by intravenous [$^{18}$F]FDG injection (7.27 ± 3.41 MBq (mean±SD), specific activity: >380.8 GBq/mg, radiochemical purity: 99.3%, Pozitron-Diagnosztika Kft. Budapest, Hungary ) for PET studies. Finally anatomical T2-weighted magnetic resonance imaging (MRI) scans were acquired for accurate atlas-based brain Volume of Interest (VOI) co-registrations (Fig. 1 b and c).

For [$^{125I}$]CLINME SPECT imaging, mice were anesthetized with isoflurane (3.5-4 % induction and then reduced to 1.52 % for maintenance of anesthesia during scanning). Before imaging 9.53±0.55 MBq (mean ± SD) [$^{125I}$]CLINME was administered intravenously following the subcutaneous injection of 14 mg/kg body weight of potassium perchlorate (Sigma-Aldrich, St. Louis, MO, USA). Anatomical T2-weighted MRI scans were also acquired. SPECT acquisition started 4 h following LPS injection (not illustrated on the time line). During the acquisitions mice were placed in prone position in a dedicated mouse bed. Body temperature was monitored and maintained at 37 ± 1 °C. Quantitative SPECT/CT images were obtained 4h

30min post injection.

*SPECT/CT*

The SPECT/CT (NanoSPECT/CT Silver Upgrade, Mediso Ltd., Budapest, Hungary) measurements were performed with multi-pinhole mouse collimators with a system SPECT resolution of 0.8 mm and the detection limit of a focal signal of 0.064 $mm^3$. The helical CT scans were acquired with 55 kV tube voltage, 500 ms exposure time, 1:4 binning and 360 projections in 3 min. In the reconstruction 0.2 mm in-plane resolution and slice thickness were set and Butterworth filter was applied. Head SPECT scanning was performed with 20 frames per cycle and termination condition of 120 s per frame in a scan range of 26.8 mm resulting in a 40 min scan. The detection peak energies were set to 24 keV in the case of I-125 and 140 keV in the case of Tc-99m with a 20% wide symmetric energy window for both nuclides. The SPECT reconstruction was performed with 0.2 mm isovoxels while the field of view was centered to the head. The results of SPECT measurements were quantified in units of radioactivity measured per unit volume (MBq/ml). The radioactivity concentrations of the segmented brain areas are presented as a ratio to the injected radioactivity concentration. Image analysis of 3D SPECT volumes of interest (VOIs) was performed with VivoQuant software (inviCRO, Boston, US).

*PET*

Dynamic PET (nanoScan® PET/MRI, Mediso Ltd., Budapest, Hungary) data acquisition was started preceding the [$^{18}$F]FDG injection and continued for 10 min post-[$^{18}$F]FDG injection in list mode. A 5 ns coincidence window and 400-600 keV energy window was applied in 94.7 mm scan range. A 3D expectation maximization (3D EM) PET reconstruction algorithm (Mediso Tera-Tomo™) was applied to produce PET images including corrections for decay, attenuation and scatter, dead time and randoms. Attenuation correction was based on automated

material map generation from MRI scout pairs [1]. After 8 iterations the reconstruction resulted in images with 0.4 mm isovoxel size and 11 time frames of different lengths. The analysed images of [$^{18}$F]FDG uptake were reconstructed from the last (11$^{th}$), 180s long timeframe. These images were used for [$^{18}$F]FDG uptake measurements.

*MRI*

Magnetic resonance imaging measurements were performed on the same integrated PET/MRI system as for the PET imaging. It is equipped with an actively shielded 450 mT/m gradient and volume coils for both reception and transmitting. As anatomic imaging a T2-weighted fast spin echo sequence was acquired with a three-dimensional acquisition scheme having a 42 mm$^2$ axial field of view and 0.3 mm in-plane resolution, the same as the slice thickness. Imaging parameters were: repetition time/echo time 2200/92.8 ms, 25 μs dwell time and two excitations resulting in a 35 min acquisition.

Brain segmentation into 3D volumes of interest (cerebrum – indicates the whole brain without cerebellum -, cerebellum, cerebral cortex and hippocampus) was performed using a connected threshold algorithm based on MRI image volumes in VivoQuant software (inviCRO, Boston, US).

*Ex vivo glutathione level measurements*

The *ex vivo* glutathione measurements were performed by a Glutathione (GSH/GSSG/Total) fluorometric assay kit (Biovision K264-100, Biovision, CA, US). After the euthanasia of animals their brains were removed and stored in cold Glutathione Assay Buffer (Biovision, CA, US) solution until the measurements. Before the tests the brains were homogenized and treated with 6 N perchloric acid (PCA) and 6 N KOH. After the elimination of excess proteins from the system by centrifugation the samples were reacted with OPA Probe (ortho-phthalaldehyde). The fluorescent signal was detected at 420 nm by a NanoDrop™ 3300 fluorospectrometer (Thermo Scientific, MA, US).

*Immunohistochemistry*

After terminal anesthesia animals were perfused transcardially with 4 % buffered paraformaldehyde (PFA). Brains were postfixed in 10 % sucrose-containing 4 % buffered PFA at 4˚C for 24 h and cryoprotected in sucrose/ PBS. After fixation 25 µm thick coronal brain sections were cut out using a sliding microtome (Leica SM2010R, Leica Biosystems Nussloch GmbH, Nussloch, Germany). After washing in PBS for 2 x 10 min at room temperature the free-floating brain sections were incubated in a PBS solution containing 2 % NDS (Normal Donkey Serum, The Jackson Laboratory 017000-121), 0.3 % Triton X100 and 20 mM sodium azide for 60 min at room temperature. Sections were incubated with rabbit anti-P2Y12 1:500 (AnaSpec Inc. Campus Drive, Fremont, CA 94555) and rat anti-mouse CD45 1:250 (Bio-Rad AbD Serotec, Kidlington, UK) primary antibodies followed by fluorochrome-conjugated donkey anti-rat Alexa594 and donkey anti-rabbit Alexa488 antisera (1:500, Thermo Fisher Scientific, Waltham, MA, USA).

After mounting and cover-slipping 6 pictures were taken of 3 serial sections (2 images/section) of each selected brain region (parietal cortex, cerebellum and hippocampus) with an Axiovert 200M inverted microscope (Zeiss). On the pictures, resting (highly ramified, P2Y12+ cells with low CD45 signal [2-5]) and activated (P2Y12+, $CD45_{low}$ ramified cells with thickened processes and enlarged body) microglia cells were counted. Blood-derived leukocytes (CD45-positive, round shape cells with predominantly perivascular location) [4, 5] were excluded from analysis.

## Results

**Fig. 1** Dynamic [$^{18}$F]FDG PET time-activity curves of the LPS treated and Control animals. (SUV: standardized uptake value). FDG uptake was calculated from frames reconstructed in the linear phase of the tracer uptake curve.

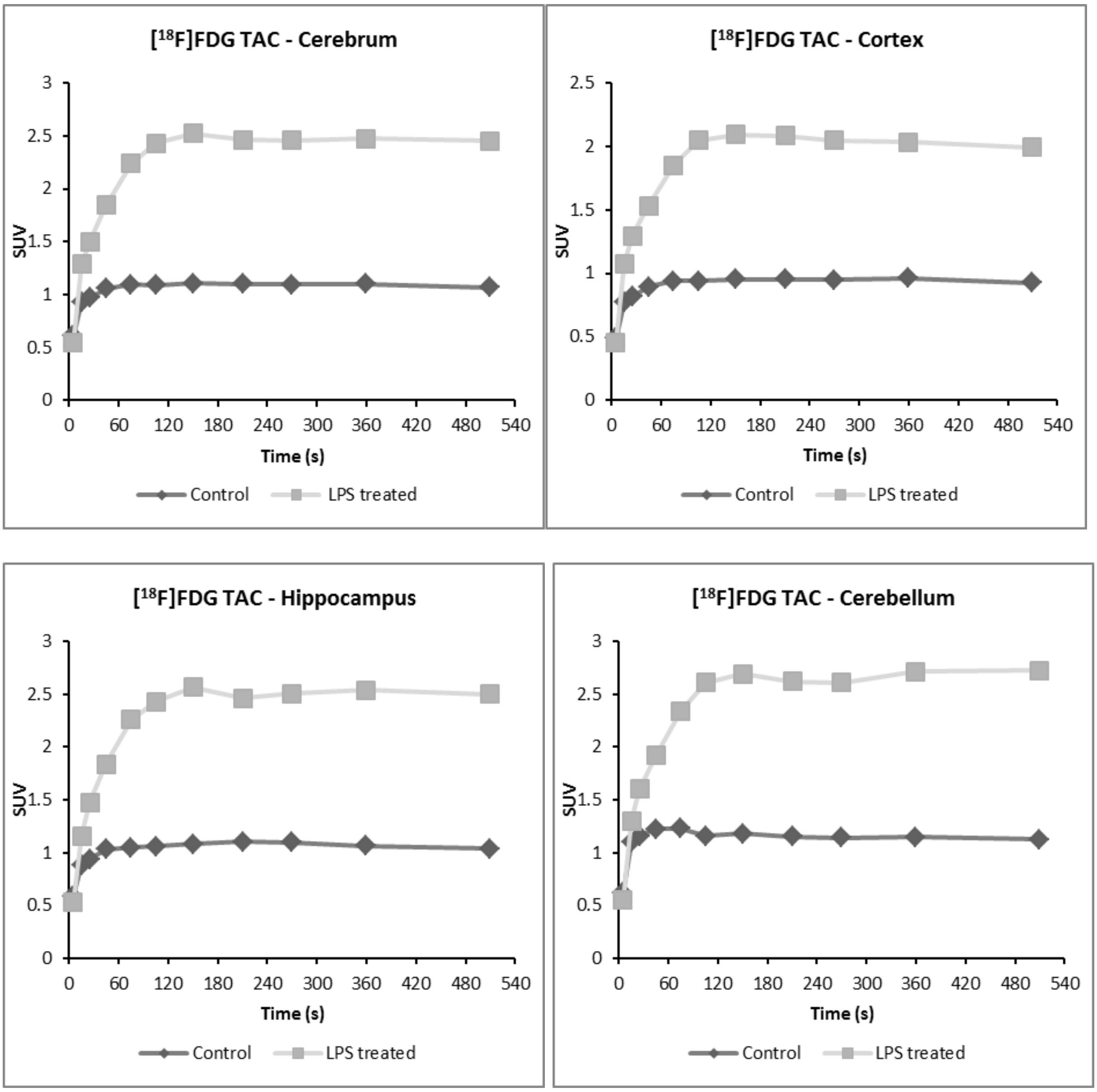


**Fig. 2.** Scatter plots of tracer uptakes in different brain regions of Control and LPS treated mice.

Linear trend lines were fitted on the data. The coefficient of determination ($R^2$) is shown above the lines.

**a. [18F]FDG SUV plotted against [99mTc]HMPAO uptake**

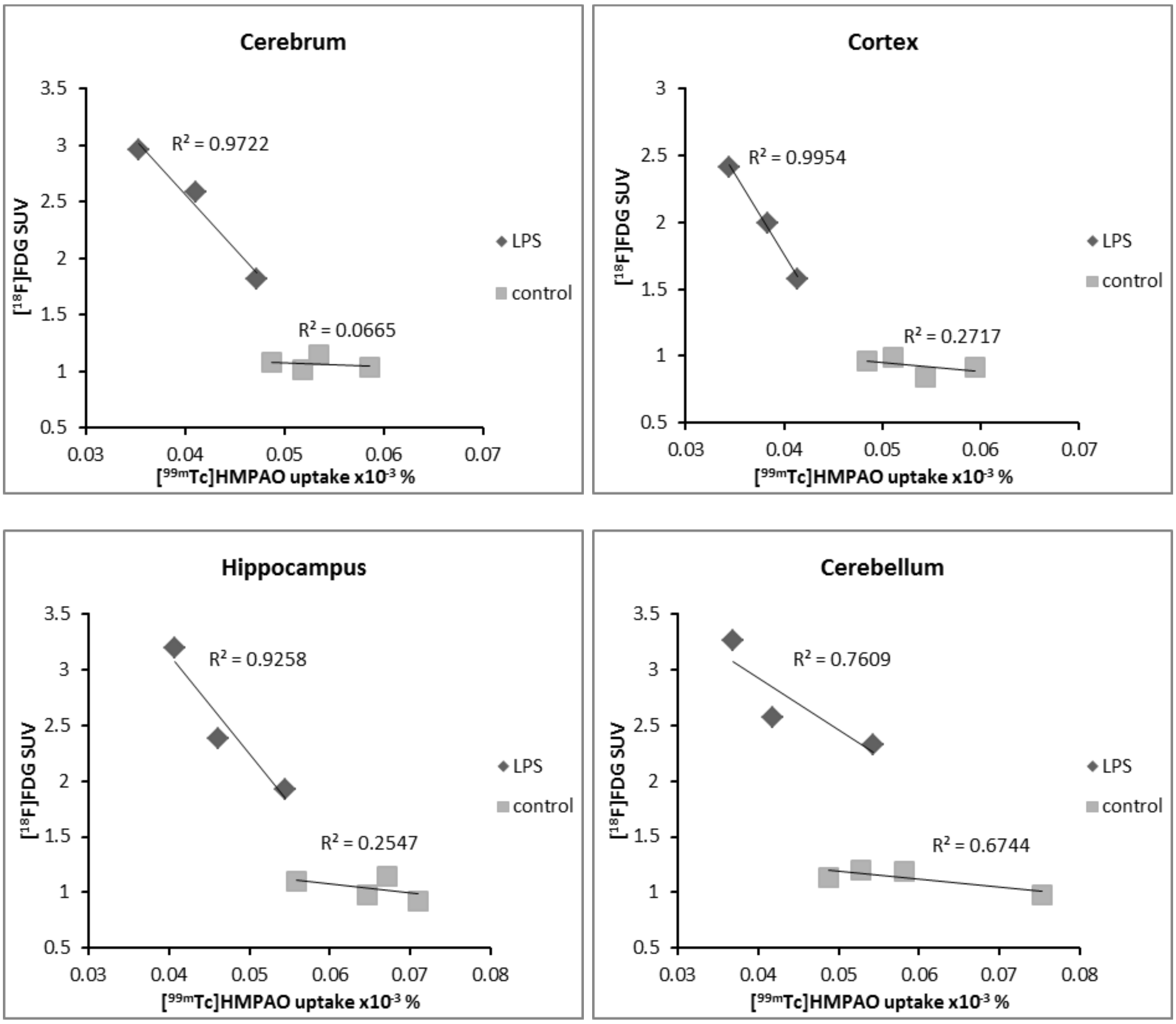

**b. [$^{18}$F]FDG SUV plotted against [$^{125}$I]Iomazenil/[$^{99m}$Tc]HMPAO uptake ratio**

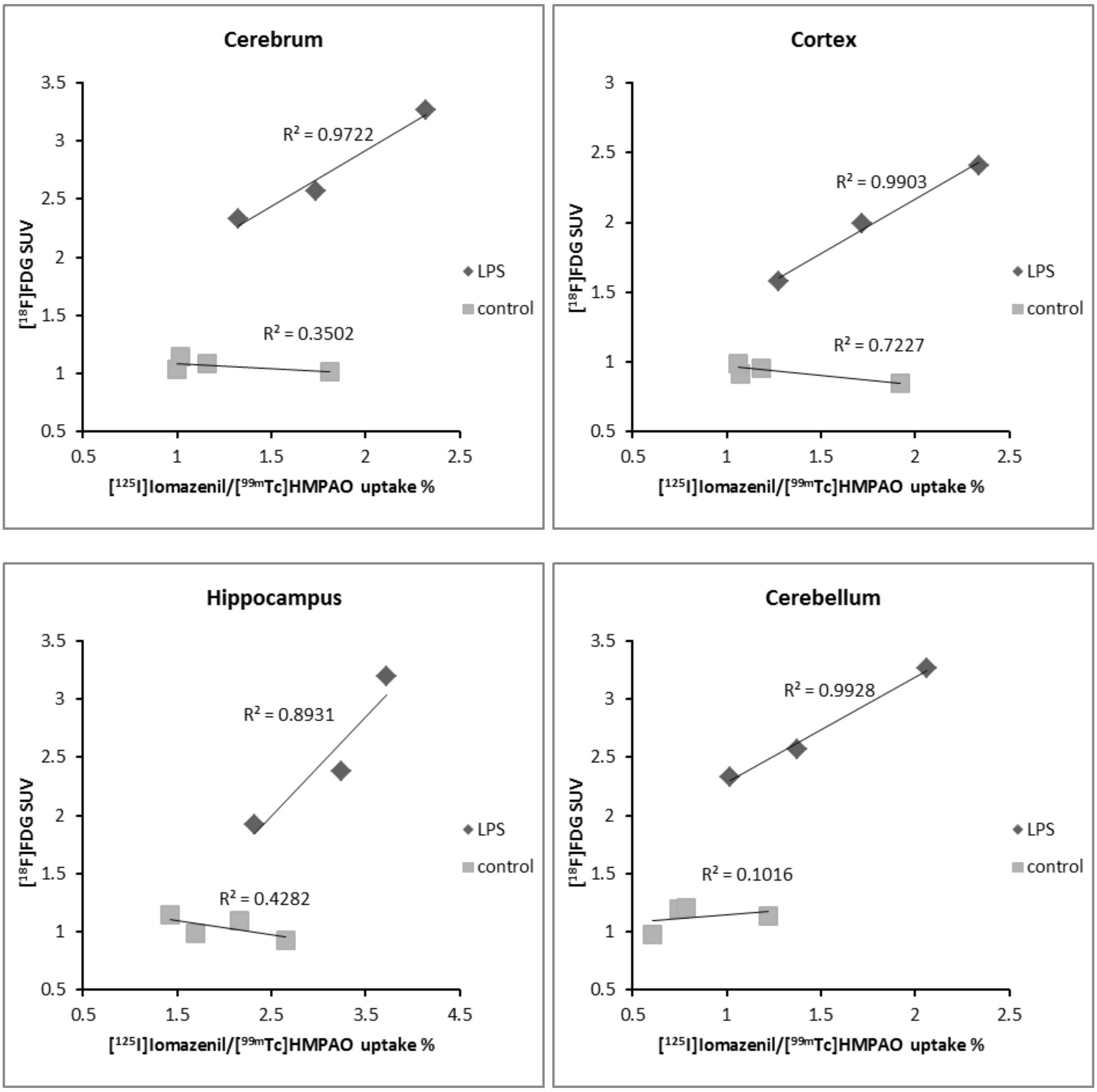

**c. [$^{125}$I]Iomazenil/[$^{99m}$Tc]HMPAO uptake ratio plotted against [$^{99m}$Tc]HMPAO uptake**

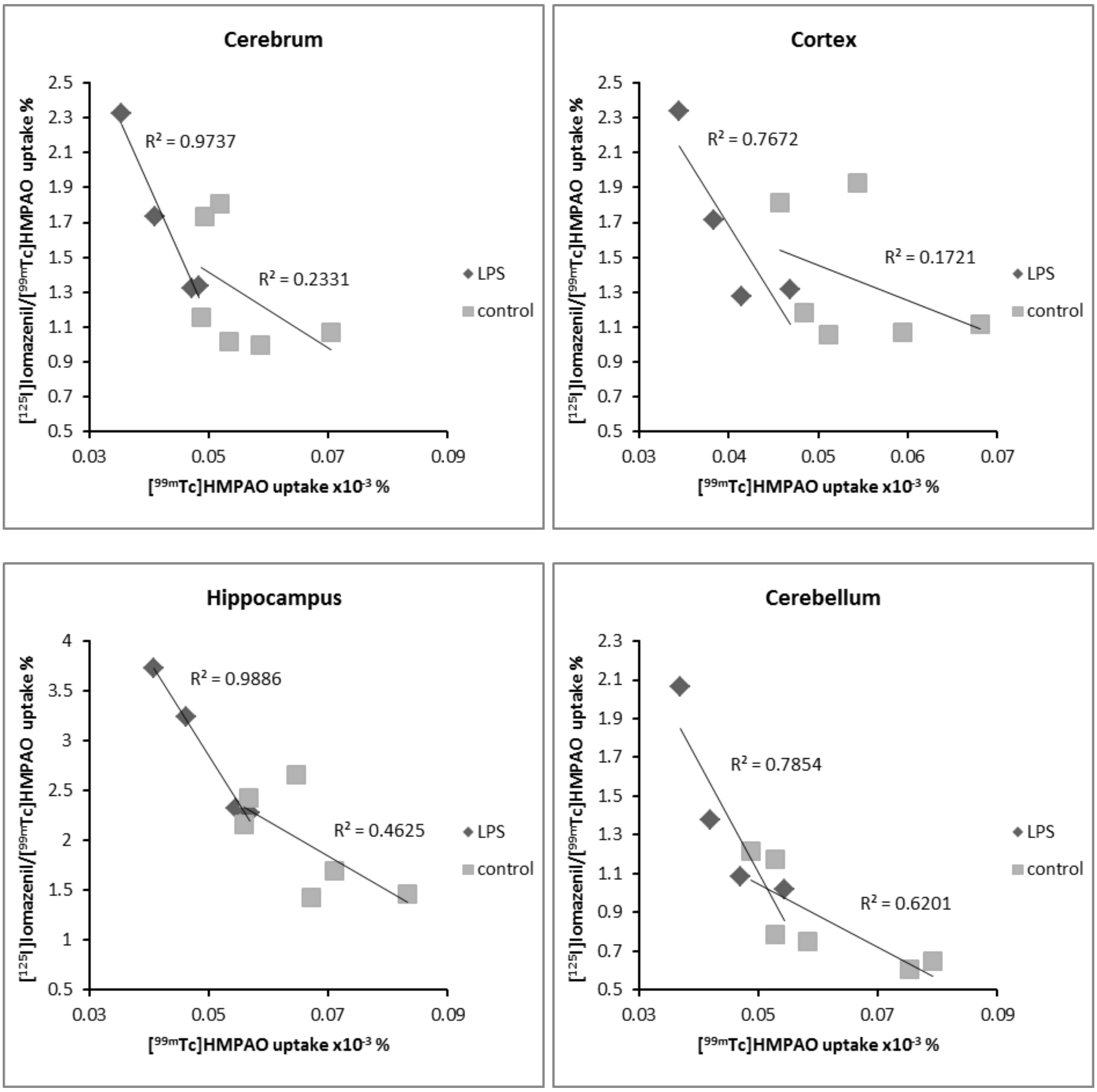


*Ex vivo glutathione level measurements*

The *ex vivo* Glutathione Fluorometric Assay Kit studies did not show any difference in the measured GSH, GSSG and total glutathione levels between the LPS-treated and the control group. The ratio of GSH/total glutathione and GSH/GSSG did not differ significantly.

1. Bandi P, Zsoter N, Koncz P, et al. (2012) Automated material map generation from MRI scout pairs for preclinical PET attenuation correction. Conf Proc IEEE Eng Med Biol Soc 2012:5351-4.

2. Kobayashi K, Yamanaka H, Fukuoka T, et al. (2008) P2Y12 receptor upregulation in activated microglia is a gateway of p38 signaling and neuropathic pain. J Neurosci 28:2892-902.

3. Swiatkowski P, Murugan M, Eyo UB, et al. (2016) Activation of microglial P2Y12 receptor is required for outward potassium currents in response to neuronal injury. Neuroscience 318:22-33.

4. Greter M, Lelios I, Croxford AL (2015) Microglia Versus Myeloid Cell Nomenclature during Brain Inflammation. Front Immunol 6:249.

5. Denes A, Coutts G, Lenart N, et al. (2015) AIM2 and NLRC4 inflammasomes contribute with ASC to acute brain injury independently of NLRP3. Proc Natl Acad Sci U S A 112:4050-5.

6. Szalay G, Martinecz B, Lenart N, et al. (2016) Microglia protect against brain injury and their selective elimination dysregulates neuronal network activity after stroke. Nat Commun 7:11499.
